# Experimental and Theoretical Studies on the Shear Performance of Concrete Beams Reinforced with Fiber-Reinforced Polymer Stirrups

**DOI:** 10.3390/ma17030593

**Published:** 2024-01-25

**Authors:** Jun Zhao, Xiaohu Bao, Shoudi Yang, Zike Wang, Hongwei He, Xiazheng Xu

**Affiliations:** 1 School of Mechanics and Safety Engineering, Zhengzhou University, Zhengzhou 450001, China; zhaoj@zzu.edu.cn (J.Z.); xiaohubao1234@163.com (X.B.); yangshoudi233@gmail.com (S.Y.); 2 Graduate School of Engineering, Kobe University, Kobe 657-8501, Japan; 3 School of Civil Engineering, Zhengzhou University, Zhengzhou 450001, China; hehongwei18866@163.com (H.H.); xuxiazheng@126.com (X.X.)

**Keywords:** FRP stirrups, concrete beams, shear performance, experiment, calculation model

## Abstract

In this paper, the shear behavior of concrete beams reinforced with FRP stirrups is studied. The shear performances of six concrete beams with a size of 150 mm × 300 mm × 3000 mm under four-point loading up to failure were experimentally analyzed. The critical parameters included the shear span to depth ratio (*λ*) and stirrup spacing (S). The test results revealed that as *λ* increased from 1 to 2, 3, and 4, the ultimate shear capacity of the beam decreases by 50.5%, 67.7%, and 69.2%, respectively. Meanwhile, as S increased from 100 mm to 150 mm and 200 mm, the ultimate shear capacity decreased by 16.1% and 44.6%, respectively. A new shear capacity calculation model of concrete beam reinforced with FRP stirrups was also proposed, which further considered the shear capacity of the FRP stirrups on the basis of the shear capacity of an RC beam without stirrups using the strut-and-tie model. Finally, the experiment and calculation results of 56 beam specimens reinforced with FRP stirrups extracted from this paper and previous studies were compared using the calculation models proposed in this paper, in order to evaluate the accuracy of these calculation models.

## 1. Introduction

To solve the corrosion problem of steel reinforcement in RC (reinforced concrete) structures and improve durability, using fiber-reinforced polymer (FRP) bars instead of steel reinforcement has become an effective method [1]. Currently, common FRP bars mainly includes glass FRP (GFRP), carbon FRP (CFRP), aramid FRP (AFRP), basalt FRP (BFRP), hybrid FRP (HFRP), and so on [2,3,4,5,6]. Among these FRP bars, GFRP bars are the most used feasible substitutes for traditional steel reinforcement due to their superior durability, mature manufacturing process, and relatively cheap price among other advantages [7]. Meanwhile, FRP bars can be used as either longitudinal reinforcement or stirrups instead of steel reinforcement in concrete structures. Because stirrups are closer to the outside of concrete structures (beams, columns, etc.), they are more susceptible to external corrosion than longitudinal reinforcement, resulting in the weakening of the transverse restraint and shear performance of the concrete member [8,9,10]. Therefore, using GFRP bars instead of steel reinforcement as stirrups will more effectively improve the durability of the whole concrete structure. Clearly, research on the performance of GFRP stirrup-reinforced concrete beams has greater significance and advantages.

Over the last 30 years, many scholars have carried out experimental studies on the shear performance of FRP-reinforced concrete beams [11,12,13,14,15,16,17]. It has been found that the shear performance of FRP-reinforced concrete beams is similar to that of concrete beams reinforced by steel, i.e., the shear capacity was also affected by the shear span to depth ratio, longitudinal reinforcement ratio, stirrup ratio and concrete strength [11]. The shear span to depth ratio was the critical factor affecting the failure mode. As the shear span to depth ratio increased, the failure mode changed from diagonal compression failure to shear compression failure, then to diagonal tension failure, and even to flexural failure [12]. Grace et al. [13] experimentally studied the failure mode and ductility of simply supported and continuous concrete beams reinforced with FRP bars. The test results showed that the type of longitudinal bars and stirrups was a key factor in the failure mode. The use of GFRP stirrups increased the deformation and deflection of the beam, and the use of GFRP longitudinal bars caused the beam failure mode to change from flexural failure to shear or flexural–shear failure. Mousavi et al. [14] studied the deflection behavior of concrete beams reinforced with GFRP longitudinal bars and considered that the bond strength of FRP bars was lower than that of steel bars, which led to increased crack depth and reduced the tension-stiffening effect, consequently resulting in greater deflection of concrete beams. Meanwhile, the elastic modulus of FRP longitudinal bars was lower than that of steel bars, so the axial stiffness of the beam after cracking was relatively small, which increased the mid-span deflection of the beam. Said et al. [15] conducted an experimental and analytical study on the shear behavior of concrete beams reinforced with GFRP bars. It was found that when the beam was damaged, the strain of GFRP stirrup was between 0.0043~0.0095. Meanwhile, the shear capacity of concrete beams reinforced with GFRP stirrups was effectively improved compared to their counterpart beams without stirrups. Ahmed et al. [16] found that there were two kinds of failure modes for concrete beams reinforced with FRP stirrups: one was the fracture failure of FRP stirrups, and the other was the crushing failure of concrete at the top flange of the beam near the loading point. Meanwhile, FRP stirrups had no effect on the initial load of shear cracking of the beam, but could enhance the contribution of concrete to its shear capacity after cracking. Similarly, the preliminary research of Hegger et al. [17] showed that when the stirrup ratio of GFRP stirrup was low, the beam failure mode was the fracture of stirrup, while when the stirrup ratio was high, it was the crushing of concrete. Recently, Maranan et al. [9] investigated the shear behaviors of geopolymer beams reinforced with continuous rectangular GFRP spirals. The test results showed that due to the higher strength of the longitudinal and transverse GFRP reinforcements compared with that of the steel reinforcement, the geopolymer concrete beams with GFRP spirals possessed obvious higher shear strength capacity than the ordinary concrete beams with steel spirals.

The shear mechanism of FRP-reinforced concrete beams is quite complex in practice, and most prediction models and design codes/standards are similar to those of steel-reinforced concrete beams [18,19,20,21,22]. According to the research of Razaqpur et al. [23], the shear capacity of FRP reinforced concrete beam is mainly composed of the following six parts:

(1) The shear resistance of uncracked concrete compression zone;
(2) Aggregate interlock;
(3) The dowel action of the longitudinal reinforcement;
(4) Arching action;
(5) Residual tensile stresses across cracks;
(6) Shear carried by the shear reinforcement.

However, different from RC beams reinforced with steel bars, the axial stiffness of the FRP-reinforced concrete beam is relatively low, and its crack width is obviously greater during failure due to the poor plastic deformation capacity and the low elastic modulus of FRP bar. Therefore, when calculating the shear capacity of a FRP-reinforced concrete beam, the friction force and the aggregate interlock force of concrete on both sides of the crack and the tensile stress of concrete between cracks can be ignored [24].

Since the 1990s, some countries have issued their own codes for the shear calculation of FRP-reinforced concrete beams. Among them, the traditional 45° truss model was adopted by the United States [18], while the variable-angle truss model based on the modified pressure field theory was adopted by Canada [19]. In each code, the shear capacity of a given beam was defined as the sum of the contribution of concrete *V*_c_ and the contribution of FRP stirrup *V*_FRP_. However, the calculation equations of current codes have some limitations. For example, the shear span ratio is generally considered to be a factor affecting the shear capacity of concrete part, but it is not considered in the American and Chinese codes. Additionally, the calculation results of the shear capacity of beams in various codes are conservative, suggesting that these codes underlying applicability to different types of concrete beams reinforced with FRP have not yet been fully explained. However, in order to ensure the applicability of the design equations to general situations, it is necessary to provide a theoretical basis for them and to determine and reasonably consider the critical parameters such as concrete strength and stirrup strength. The purpose of this paper is to solve this problem. In addition, many scholars have also proposed some new more complex shear models using artificial learning methods, such as the genetic algorithm theory [25], the rigid body rotation shear model [26], and so on.

However, to the best of the authors’ knowledge, since FRP reinforced concrete beams are rarely used in actual structures and the calculation of their shear capacity is more complicated, there is limited research on investigations into the shear properties of concrete beams reinforced with types of FRP stirrups. Therefore, comprehensive experiments and theoretical analyses were carried out to investigate the shear capacity of FRP-reinforced concrete beams in this study. The rest of this article is organized as follows: Section 2 introduces in detail the four-point bending test of six reinforced concrete (RC) beams reinforced with FRP stirrups. Section 3 completely discusses the effects of shear span to depth ratio (*λ*) and stirrup spacing (*S*) on the deflection, strain, bearing capacity, crack form, and failure mode of FRP-reinforced concrete beams. In Section 4, the calculation formula of the shear capacity of concrete beams without web reinforcement is established based on the strut-and-tie model, and further, a new shear capacity calculation model of concrete beams reinforced with FRP stirrups is proposed by using superposition with the bearing capacity of the FRP stirrup. Finally, the accuracy of the proposed model in this study was evaluated through comparing the test results of 56 beam specimens extracted from this study and previous studies, with the results calculated using models from American, Canadian, and Chinese codes, alongside this study.

## 2. Experimental Program

### 2.1. Beam Specimen Design

**Figure 1:**
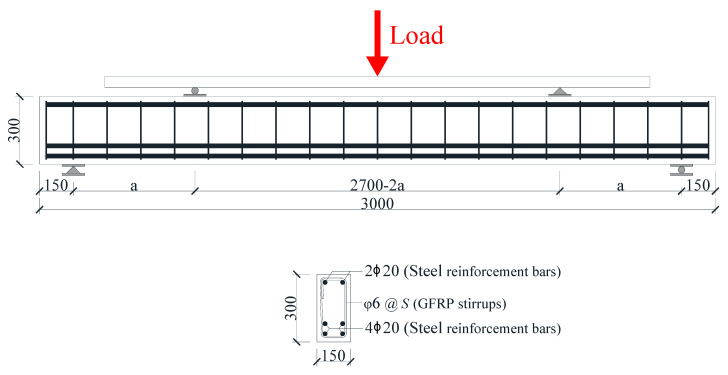
Configuration and details on concrete beams reinforced with GFRP stirrups (dimensions in mm).

A total of 6 concrete beams reinforced with GFRP stirrups were tested. The dimensions of all beam specimens were designed to be 150 mm in width, 300 mm in depth, 3000 mm in length and 2700 mm in span [27], as shown in Figure 1. Steel bars with diameter of 20 mm were adopted as the longitudinal reinforcement, and GFRP bars with diameter of 6 mm were adopted as the stirrups. Each beam specimen contained six longitudinal steel bars, two of which were placed on the top, and the other four were arranged in two rows at the bottom of the beam. The concrete cover of each beam was uniformly 20 mm. In this study, four shear span to depth ratios (*λ* = 1, 2, 3 and 4) and three stirrup spacings (*S* = 100, 150 and 200 mm) were designed as the parameters influencing the shear capacity of beams. Each beam specimen was identified by a code starting with “B”. More specific parameters and details of the beams are listed in Table 1.

### 2.2. Materials

**Figure 2:**
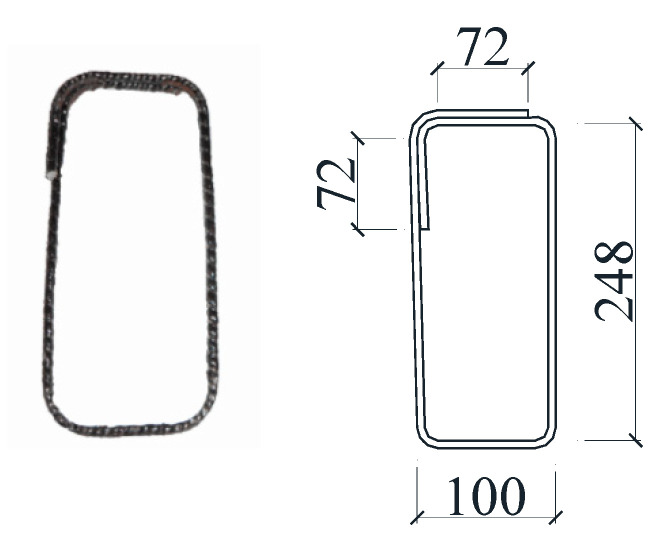
Photograph and schematic diagram of GFRP stirrup (dimensions in mm) [27].

In this study, ordinary concrete with a compressive strength grade of C30 was used, and the standard value of the cubic compressive strength of concrete *f_cu_* was tested to be 33.00 MPa, which was converted into a cylinder compressive strength *f_c_*′ of 26.07 MPa (*f_c_*′ = 0.78*f_cu_*). HRB400 steel bars of diameter of 20 mm were used as longitudinal reinforcements, and GFRP stirrups of diameter of 6 mm were used as shear reinforcements to reinforce the concrete beams. A photograph and schematic diagram of the GFRP stirrups are shown in Figure 2 [27]. According to ASTM D7205/D7205M-06 [28], the tensile properties of GFRP bars were determined using straight GFRP bars with a diameter of 6 mm, and were made of the same material as the GFRP stirrups. The tested tensile strength and elastic modulus of GFRP bars were 716.3 MPa and 55.6 GPa, respectively. The detailed tensile test results of GFRP bars are shown in Table 2.

### 2.3. Beam Specimen Preparation

**Figure 3:**
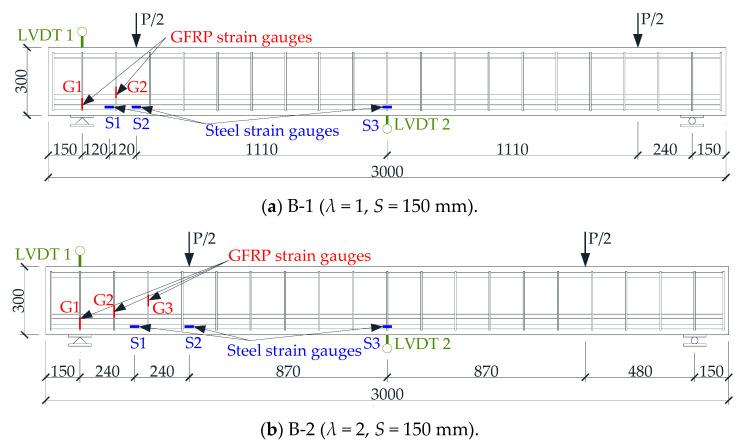

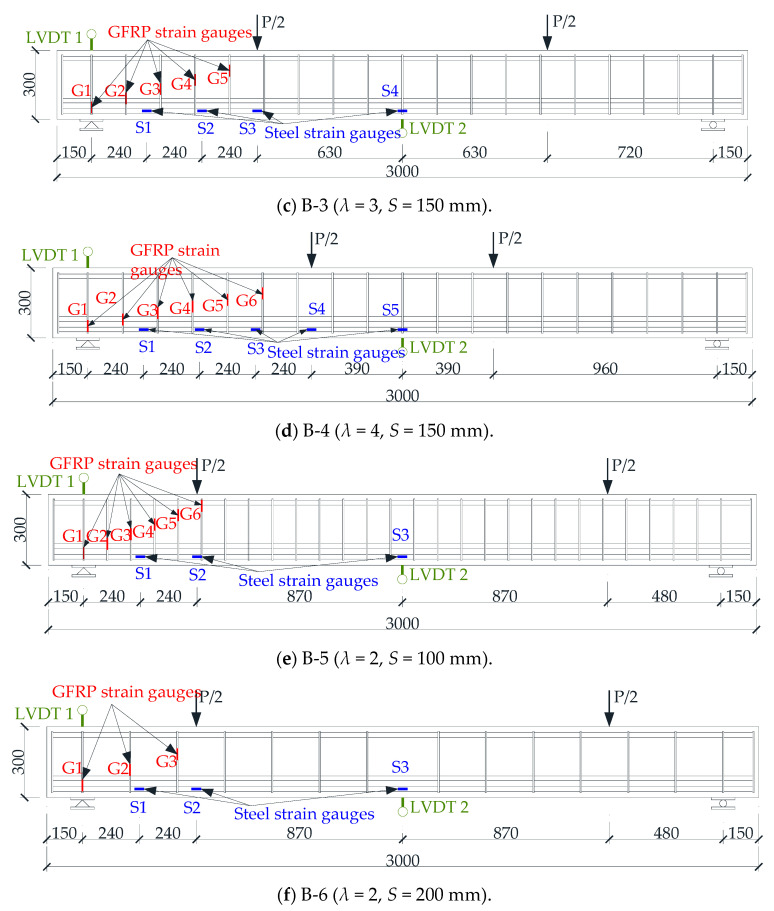
Schematic diagram of layout of strain gauges and LVDTs.

**Figure 4:**
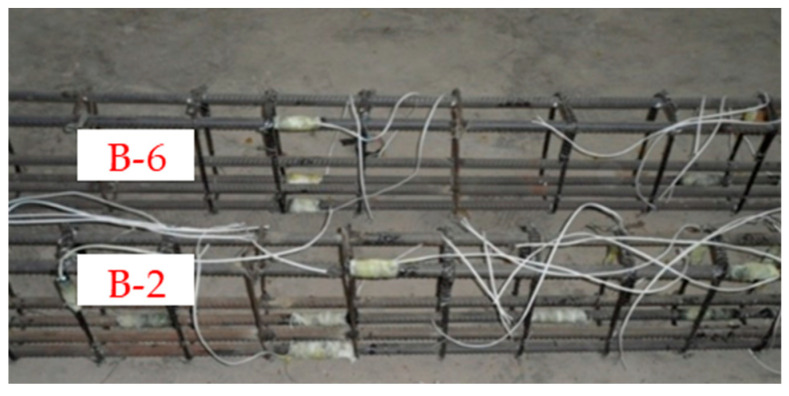
Reinforcing cage consisting of steel bars and GFRP stirrups with strain gauges pasted. Note: The top specimen is B-6 (*λ* = 2, *S* = 200 mm); the bottom one is B-2 (*λ* = 2, *S* = 150 mm).

In order to accurately measure the deformation of the beam specimen and the strain of the stirrup and longitudinal reinforcement during the loading process, strain gauges were pasted to the designated positions of the GFRP bars and longitudinal steel bars before the concrete was poured, and linear variable differential transformers (LVDTs) were placed at the designated positions against the concrete surface before the test loading. The specific arrangement of strain gauges and LVDTs for each beam specimen is shown in Figure 3. Taking the vertical central axis in the middle of the beam span as the axis of symmetry, all the LVDTs and strain gauges were symmetrically distributed on the left and right sides of the beam. For simplicity, only the LVDTs and strain gauges on the left half span of the beam were marked. A reinforcing cage consisting of GFRP bars and longitudinal steel bars with the strain gauges pasted is shown in Figure 4.

### 2.4. Test Set-Up and Loading Program

**Figure 5:**
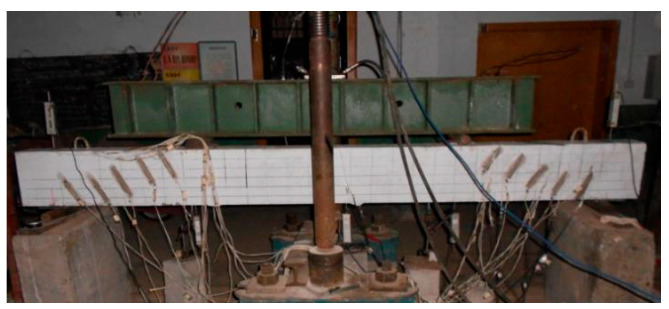
Experimental set-up of beam specimen.

The beam specimen was simply supported on reinforced concrete piers with a span of 2700 mm, as shown in Figure 5. The loading procedure mainly included two phases, i.e., preloading and formal loading, according to GB 50152-92 [29]. Before each formal loading, the specimen beam was applied with a preload load not exceeding 70% of the calculated value of the cracking test load, and then we checked whether the strain gauge and displacement meter worked normally.

The formal loading was divided into the following parts:

(1) At the beginning, the load level of each level was 10 kN and maintained for 10 min.
(2) When the load was close to the cracking of concrete, the load level was changed to 5 kN per level. When the first crack appeared in flexural zone of beam between two loading points, the load value was recorded as the first crack load *V_cr_*.
(3) The load level was restored to 10 kN per level after the first crack appeared. When the first oblique crack appeared in the flexural compression zone, the load value was recorded as the first diagonal shear crack *V_scr_*.
(4) When the maximum crack expands to 1.5 mm, the load value of the beam specimen was recorded as the shear capacity of experiment *V_exp_*.

## 3. Results and Discussions

The test results of the mid-span deflection, crack development, and failure mode of all the beam specimens are summarized in Table 3. The influence of shear span to depth ratio (*λ*) and stirrup spacing (*S*) on the shear performances in terms of the load deflection behavior, load–strain behavior, shear capacity, crack patterns, and failure modes of the tested beams were discussed in turn, as follows.

### 3.1. Shear Force–Deflection Response

**Figure 6:**
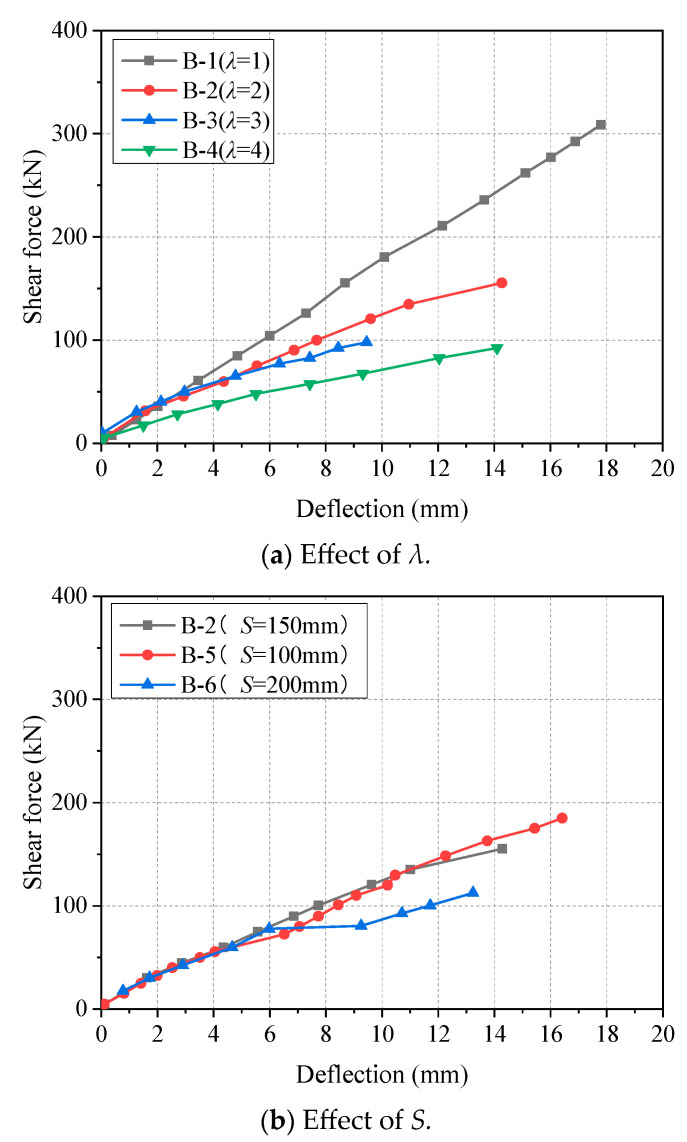
Shear force–deflection curves at midspan of beam specimens.

The effects of shear span to depth ratio (*λ*) and stirrup spacing (*S*) on the shear force-deflection responses at midspan of the beam specimens are shown in Figure 6. As is found, before the first crack, the shear-deflection behavior of all beams was nearly linear. After cracking, the effective concrete area decreased, and the moment of inertia decreased, so that the shear force–deflection curve of the beams was still linear, but the stiffness was reduced [30]. Figure 6a revealed that the bending moment increases rapidly with the increase in *λ*, and the deformation of the beam also increases [31]. It is worth noting that in Figure 6b the deflection of specimen B-6 suddenly increased at 78 kN. This is due to an insufficient stirrup ratio causing some stirrups to reach the limit deformation earlier and brittle fracture. That is to say, the inhibition of GFRP developing into inclined cracks in concrete improves the bearing capacity and ductility of beam specimens. On the other hand, for B-2 and B-5, the slope remains linear, revealing that the contribution of GFRP stirrups to the beam shear capacity remains at a high level. In Summary, GFRP, as a kind of stirrup, exerts its high strength and high ductility to improve the strength and deformation capacity of concrete beams.

### 3.2. Shear Force–strain Response

**Figure 7:**
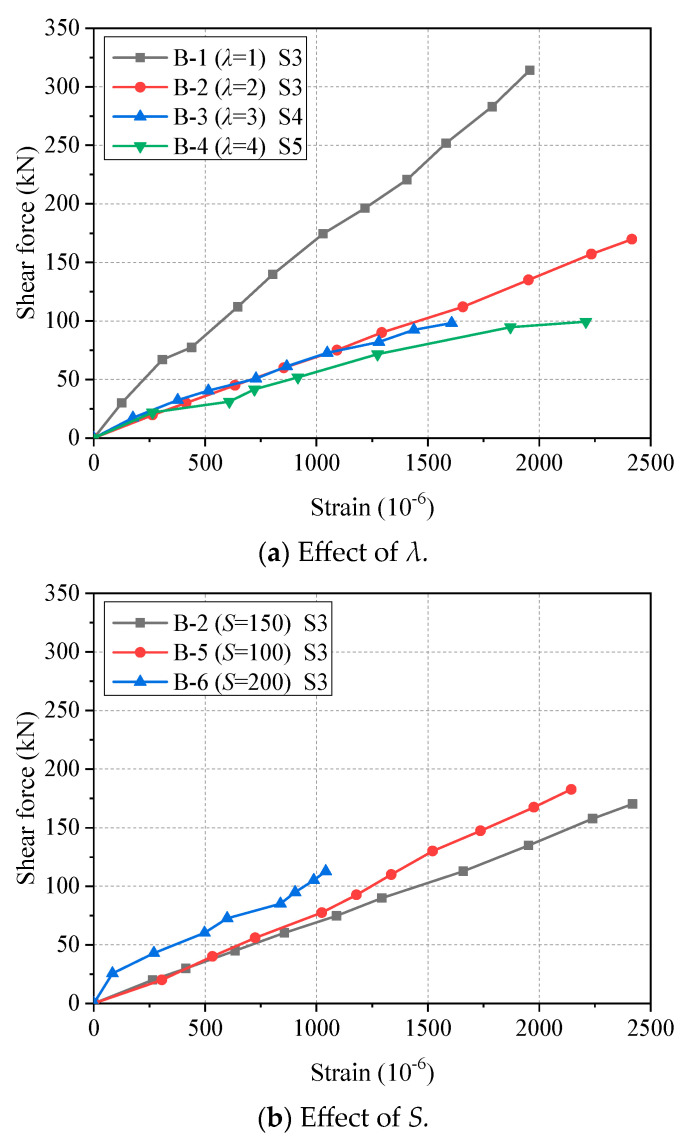
Shear force–strain curves at midspan of longitudinal steel bars.

**Figure 8:**
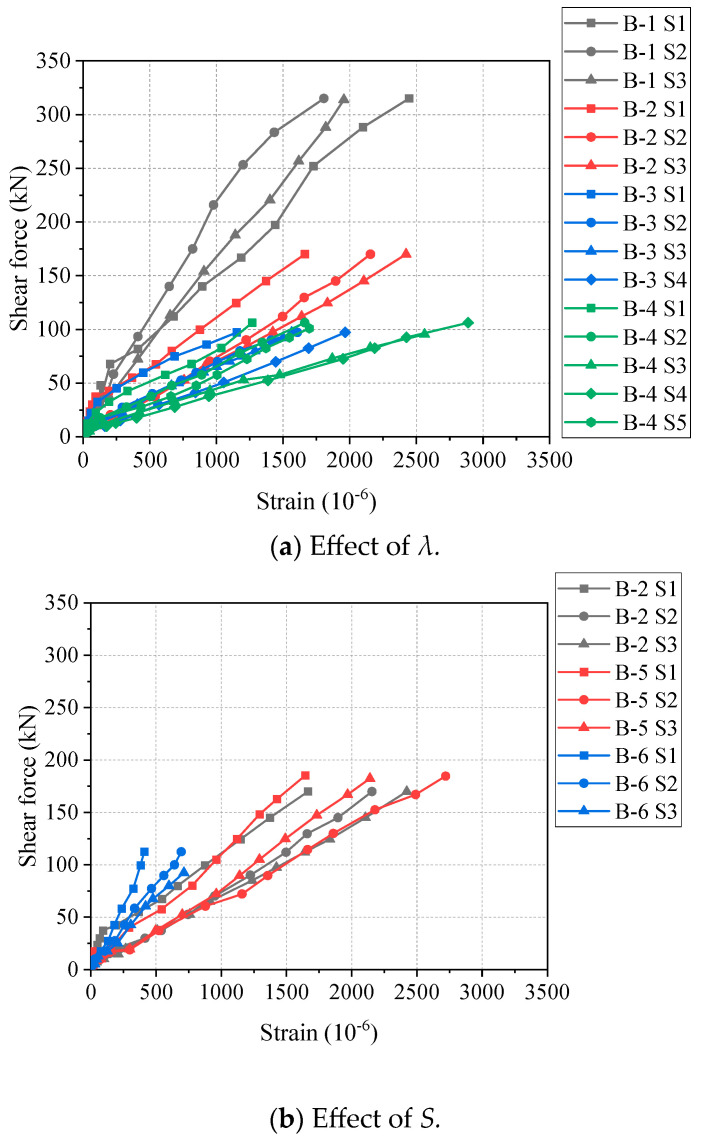
Shear force–strain curves at different positions of longitudinal steel bars.

The relationships between the shear force and the strain of longitudinal bars of all beam specimens are shown in Figure 7. It can be found that all beams have a similar shear force–strain linear relationship. As shown in Figure 7a, with the increase in *λ*, the slope of the curve decreases. Since the larger *λ* leads to the larger bending moment in the purely bending part of the beam, the strain of the longitudinal reinforcement will be greater under the same shear force. As shown in Figure 7b, the influence of *S* on the strain of steel longitudinal reinforcement is not significant, which is similar to the results of previous tests [31]. In addition, the shear force–strain curves at different positions of longitudinal bars are shown in Figure 8. Generally, the slope of the curve at the position closer to the support is larger. This is because when the position is closer to the support point, the shear force of the beam is larger.

**Figure 9:**
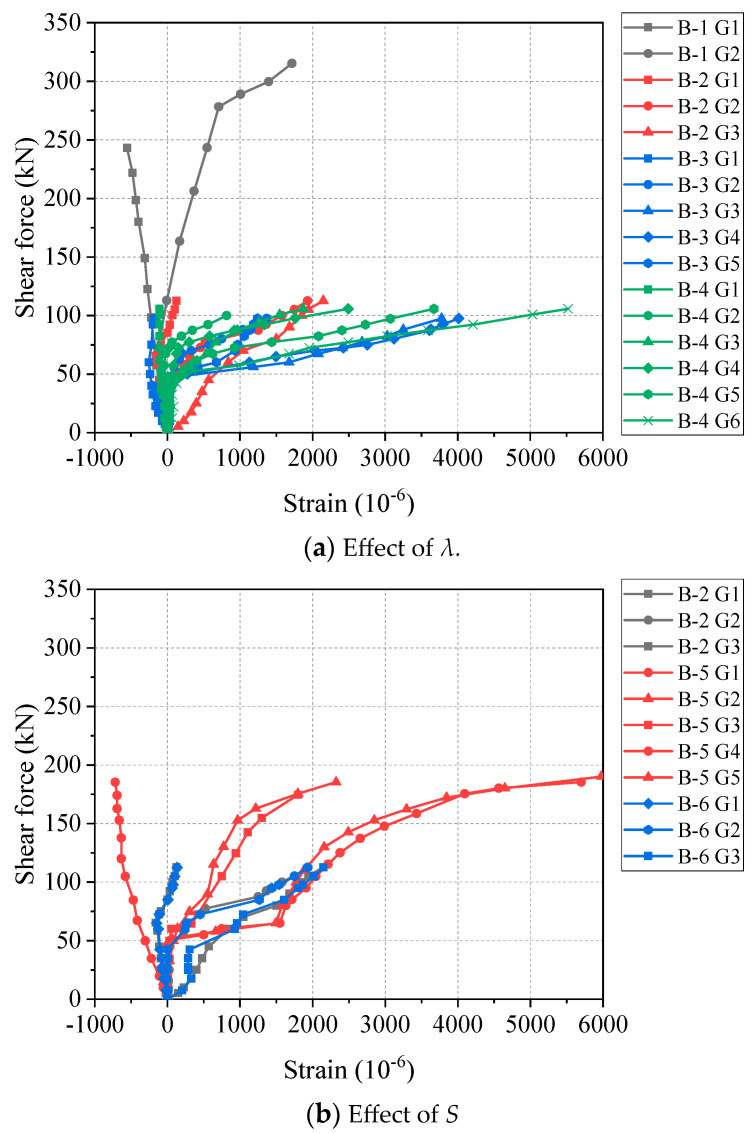
Shear force–strain curves at different positions of GFRP stirrups.

Figure 9 illustrates the relationship between the shear force and strain of the GFRP stirrups at different positions between the loading point and support of the beam. All the curves in Figure 9 can be divided into two types according to the different positions: (1) Near the support point (G1), the strain increased slowly with a negative slope as the shear force increased. This is because the stirrups and concrete at the support were locally compressed; (2) for the stirrups between the support and the loading point, the strain suddenly increased after the appearance of cracks. This indicated that the shear contribution of the stirrups increased. The larger changes of the slope illustrate the effectiveness of GFRP stirrups in improving the shear capacity of beams. It is worth noting that the maximum strain of the stirrups of each beam is between 0.002–0.006. Their average value is 0.004, which is consistent with the ultimate strain of 0.004 for stirrups in ACI 440.1R-15 [18].

### 3.3. Shear Capacity

**Figure 10:**
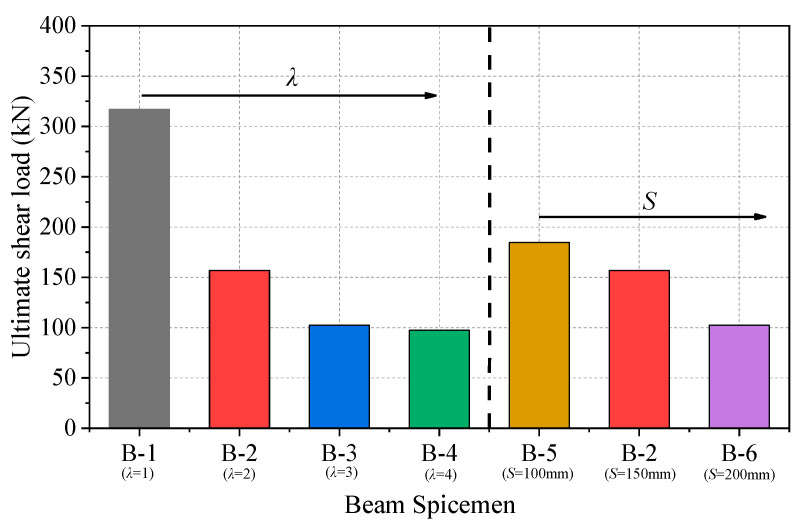
Ultimate shear force of beam specimens with different *λ* and *S* values.

Figure 10 shows the variation in the ultimate shear force of the beam specimens with *λ* and *S*. With the increase in *S* and *λ*, the ultimate shear force tended to decrease. On the one hand, as *λ* increased from 1 to 2, 3, and 4, the ultimate shear force of the beam decreased by 50.5%, 67.7%, and 69.2%, respectively. The increase in *λ* led to a decrease in the critical shear crack angle, so the bearing capacity of the corresponding section decreased, and the shear capacity decreased accordingly. On the other hand, as *S* increased from 100 to 150 and 200, the ultimate shear force of the beam decreased by 16.1% and 44.6%, respectively. This was because the decrease in *S* means denser stirrups, which confined concrete and limited the development of the cracks of the beam.

### 3.4. Crack Patterns

**Figure 11:**
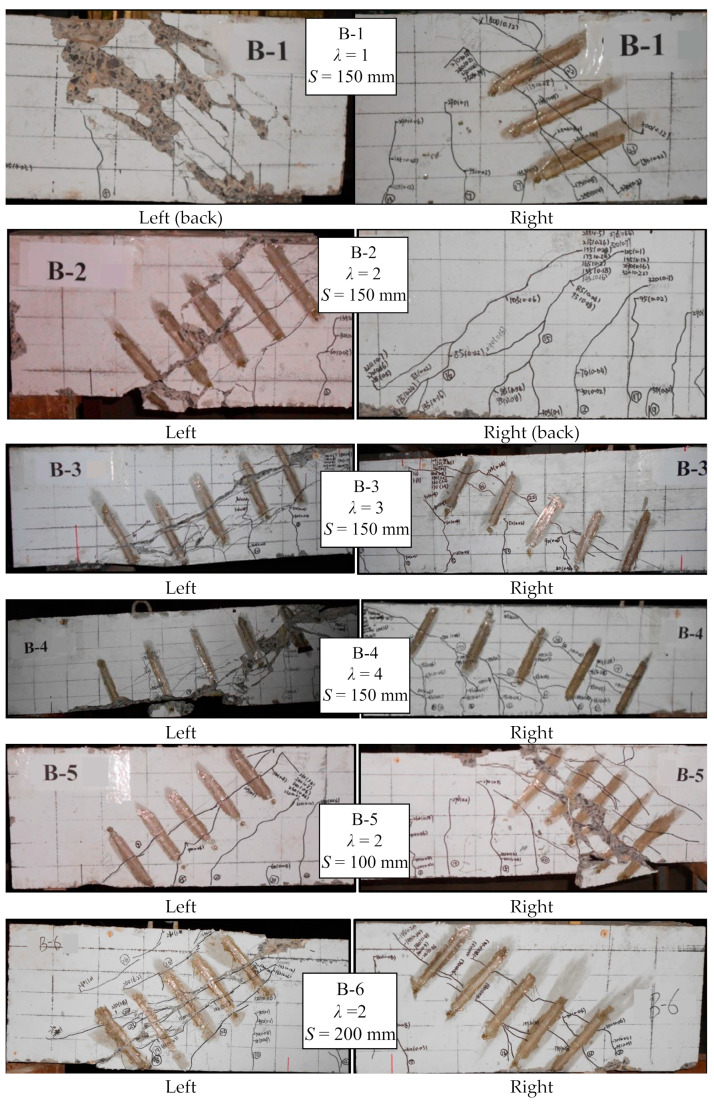
Crack patterns and failure modes.

The crack patterns of the beam specimens are shown in Figure 11. Similar crack development can be observed in B-1~B-6. As is found in Table 3, the first cracks of B-1 to B-6 were found under the load of 15 kN~35 kN, and they all appeared in the pure bending zone. The B-1 gave the first crack load of 35 kN. The first crack loads of the B-2, B-3 and B-4 were 43%, 39% and 57% smaller than that of B-1 due to increase in shear span to depth ratio. A comparison was made between B-5, B-2 and B-6, S increased from 100 to 150 and 200, and the load for the first crack was basically unchanged. The results showed that the shear span to depth ratio had an evident influence on the first crack load.

The crack development of the beam specimens can be roughly divided into the following three types: (1) For B-1, with the increase in load, several oblique shear cracks appeared in the beam abdomen (e.g., middle zone) between the loading point and the support. The oblique cracks were many and dense when the beam was damaged. (2) For B-2, B-5 and B-6, with the increase in load, many shear inclined cracks appeared in the beam. The beam finally failed along one of the main cracks and the concrete fell off. (3) For B-3 and B-4, the oblique crack in the beam rapidly extended to the point of concentrated load, and formed a long main oblique crack from the loading point to the support.

**Figure 12:**
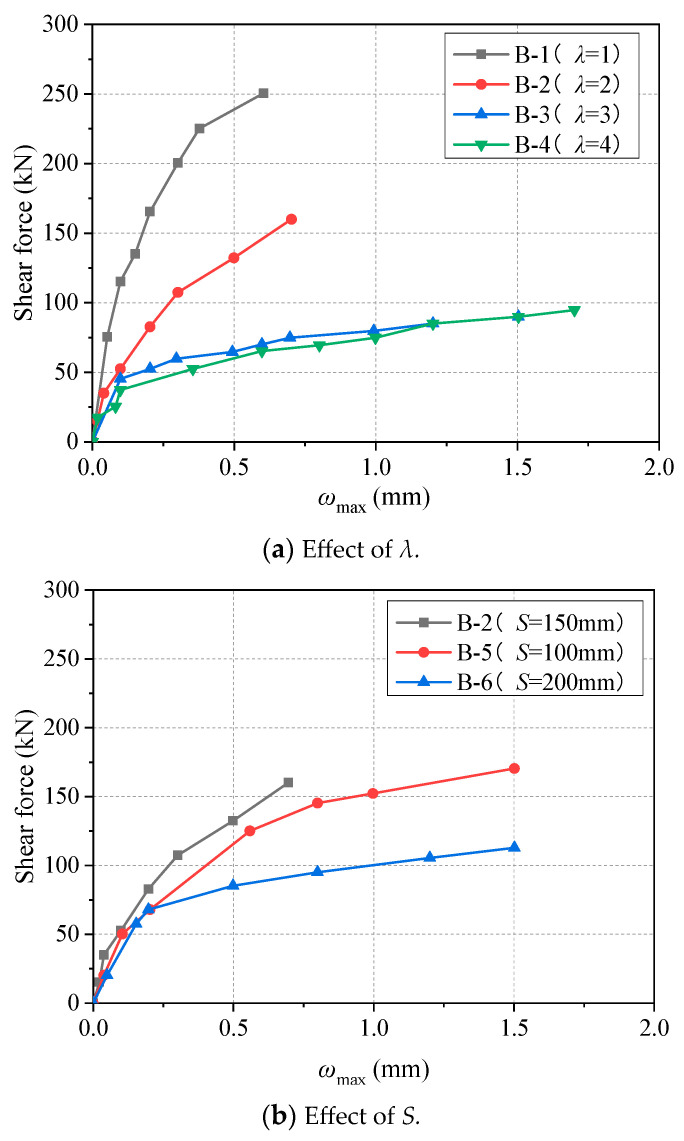
Relationship curves between the maximum diagonal crack width and shear force of all beam specimens.

The relationship curves between the maximum diagonal crack width of beam *ω_max_* and the shear force are shown in Figure 12. The *ω_max_* increases with the increase in shear force, and the slope of the curve increases with the increase in shear force. As can be seen from Figure 12a, the slope of the curve increases faster with the increase in *λ*. As can be seen from Figure 12b, the curve increases linearly at the beginning, and there is an obvious sudden increase before failure, and the slope of the curve will increase faster with the increase in *S*. Therefore, *λ* and *S* are both important factors affecting crack development.

### 3.5. Failure Mode

Compared with previous studies by Said et al. [12,15], we found that there are a total of three types of failure from B-1~B-6., i.e., diagonal compression failure (DC), shear compression failure (SC), and diagonal tension failure (DT). The detailed failure mode of each beam is given in Table 3. Among them, B-1 suffered DC, B-2, B-5 and B-6 suffered SC, and B-3 and B-4 suffered DT.

As we found, *λ* was the critical factor affecting the failure mode. When *λ* was relatively small (*λ* ≤ 1), the diagonal crack extends from near the support point to the loading point. With the increase in load, the crack gradually became horizontal and eventually joined, and finally, the GFRP stirrups and concrete located in the main inclined crack were broken. This was similar to the failure mode of deep beams reinforced with GFRP bars described by Andermatt [32]. When 1 < *λ* < 3, a crack first appeared at the loading point, and then extended from the support point to the loading plate point to form a critical diagonal crack. The beams could carry the load after the critical diagonal crack formed. Its failure was characterized by the crushing of the concrete near the loading point. When λ ≥ 3, the major diagonal curved cracks appeared in the shear span section of the beam, and developed from the support point to the loading point with the increase in load. As the load continued to increase, parallel cracks formed in the bottom of the shear span section and intersected with the diagonal cracks. Finally, the characteristic of shear tension failure was evident in the concrete cover, and some small concrete pieces inside the stirrups were spilled out.

## 4. Calculation Model of the Shear Capacities of Concrete Beams Reinforced with FRP Stirrups

### 4.1. Model Establishment

**Figure 13:**
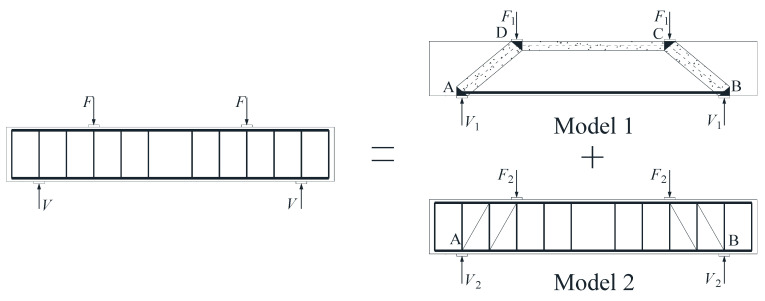
Shear capacity model of concrete beams reinforced with FRP stirrups.

After the beam specimen appeared oblique cracks, the beam mainly bore the shear force through the tension of the stirrups and some longitudinal reinforcements and the compression of the concrete. As shown in Figure 13, the shear capacity model of the beam was composed of Model 1 and Model 2, which were used to describe beams without a web reinforcement beam and with only stirrups, respectively.

### 4.2. Shear Capacity of Concrete Beams without Web Reinforcement: V_1_

Xiong et al. [33] summarized and put forward the calculation model of shear capacity of concrete beams without web reinforcement. As we found, for concrete beams without web reinforcement, there was an obvious strut-and-tie model when the oblique section of the beam was damaged by shear [34]. As shown in Model 1 in Figure 13, the strut-and-tie model consisted of struts, ties, and nodes. The compressive force was carried by the portions of the concrete between inclined cracks known as struts, and the tension force was carried by the bottom longitudinal reinforcements known as ties. Nodes were the locations where the axes of the struts, ties, and concentrated forces intersected.

#### 4.2.1. Equilibrium Equation

**Figure 14:**
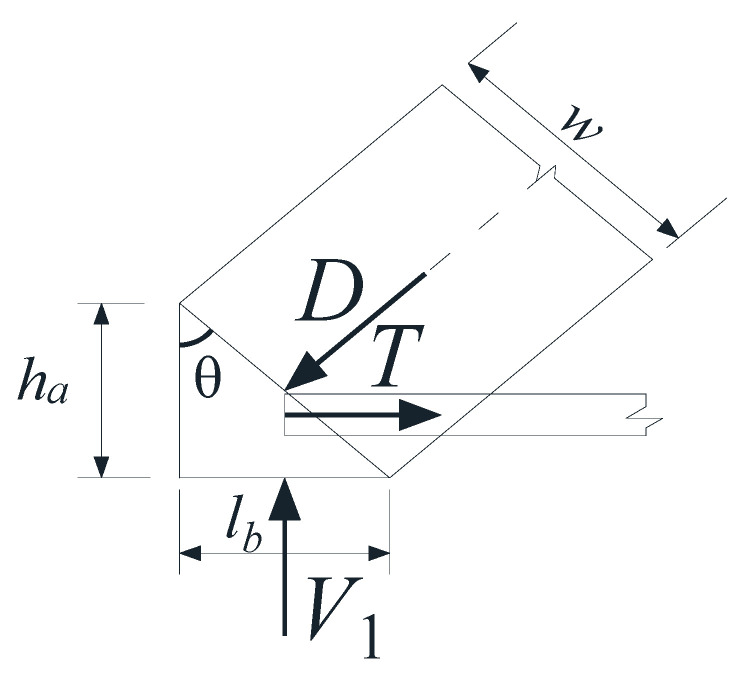
Force and geometric diagram of point A in Model 1.

A free-body diagram of a segment of point A from the left support is shown as shown in Figure 14. For equilibrium, the stress of node A of Model 1 must be balanced:

(1) $$V_{1}=D\sin\theta$$

(2) $$T=V_{1}\cot\theta$$

where *D* is the compression capacity of strut.

(3) $$D=f_{2max}bw$$

where *f*_2*max*_ is the compressive stress of strut, and *θ* is the angle between strut and beam transverse axis, referring to the literature [35].

(4) $$\theta =\arctan\left[\left(h_{0}-0.5d_{a}\right)/a\right]$$

where *d_a_* is the height of a concrete horizontal compression bar between nodes C and D. According to reference [36],

(5) $$d_{a}=0.4kh_{0}$$

where *k* is the height coefficient of concrete compression area, and can be expressed as follows:

(6) $$k=\sqrt{{\left(n\rho \right)}^{2}+2n\rho }-n\rho$$

where *ρ* is the longitudinal reinforcement ratio. *n* is the elastic modulus ratio of longitudinal reinforcement (*E_s_*) to the elastic modulus of concrete (*E_c_*), i.e., $n=E_{s}/E_{c}$ and $E_{c}=4730\sqrt{f_{c}^{\prime }}$ [37].

#### 4.2.2. Constitutive Equation

When *f*_2max_ increases to the compressive strength of concrete with oblique cracking *βf_c_*′, *D* reaches its limit, which is equivalent to Model 1 reaching its shear capacity.

**Figure 15:**
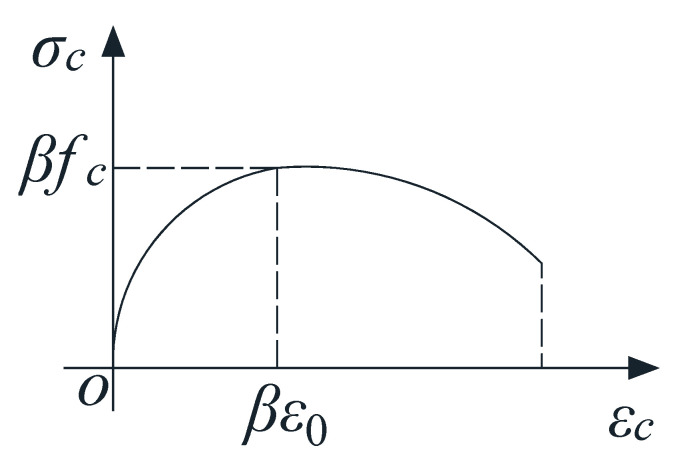
Stress–strain curve of softened concrete.

According to previous studies [38], the stress–strain relationship in the principal compressive stress direction of softened concrete is as follows:

(7) $$\begin{array}{ll} \sigma_{c}=\beta f_{c}^{\prime }\left[\frac{2\varepsilon_{c}}{\beta \varepsilon_{0}}-{\left(\frac{\varepsilon_{c}}{\beta \varepsilon_{0}}\right)}^{2}\right] & \left|\varepsilon_{c}\right|\leq \left|\beta \varepsilon_{0}\right|\\ \sigma_{c}=\beta f_{c}^{\prime }\left[1-{\left(\frac{\varepsilon_{c}/\varepsilon_{0}-\beta }{2-\beta }\right)}^{2}\right] & \left|\varepsilon_{c}\right|>\left|\beta \varepsilon_{0}\right| \end{array}$$

where *f_c_*′ is the specified compressive strength of concrete; *β* refers to the softening effect coefficient of the compressive strength of cracked concrete, *ε*_0_ is the maximum compressive strain of softened concrete (recommended to be taken *ε*_0_ as 0.002), *ε_c_* is the compressive strain of softened concrete, and *σ_c_* is the compressive stress of softened concrete. The stress–strain curve of softened concrete corresponding to Equation (7) is shown in Figure 15.

Vecchio and Collins obtained the following simplified calculation formula in their research [39]:

(8) $$\beta =\frac{1}{0.80+170\varepsilon_{1}}\leq 1.0$$

where *ε*_1_ is the principal tensile strain of concrete in the direction perpendicular to strut.

Therefore, *f*_2*max*_ can be expressed as follows:

(9) $$f_{2max}=\beta f_{c}^{\prime }=\frac{f_{c}^{\prime }}{0.80+170\varepsilon_{1}}\leq 0.85f_{c}^{\prime }$$

#### 4.2.3. Compatibility Condition

**Figure 16:**
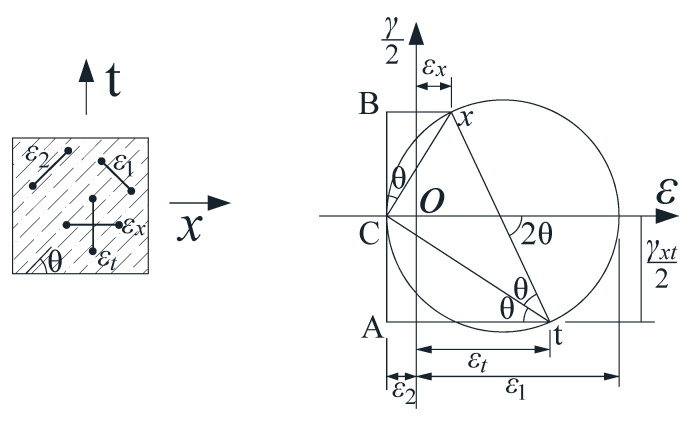
Compatibility conditions of a cracked element.

The strain relationship of beam elements after concrete cracking can be represented by Mohr’s circle [36], and the compatibility conditions for cracked elements are shown in Figure 16. The following formula can be derived from Mohr’s circle:

(10) $$\frac{\gamma_{xt}}{2}=(\varepsilon_{x}-\varepsilon_{2})\cdot \cot\theta$$

(11) $$\frac{\gamma_{xt}}{2}=(\varepsilon_{t}-\varepsilon_{2})\cdot \tan\theta$$

where *ε_x_* is the longitudinal strain of the element, *ε_t_* is the transverse strain of the element, *γ_xt_* is the shear strain of the element, and *ε*_2_ is the principal compressive strain of the strut.

The relationship between strains can be obtained from Equations (10) and (11):

(12) $$\frac{\left(\varepsilon_{x}-\varepsilon_{2}\right)}{\left(\varepsilon_{t}-\varepsilon_{2}\right)}=\tan^{2}\theta$$

The following relationship is obtained from the first strain invariant:

(13) $$\varepsilon_{1}+\varepsilon_{2}=\varepsilon_{x}+\varepsilon_{t}$$

We obtain the following from Equations (8) and (9):

(14) $$\varepsilon_{1}=\varepsilon_{x}+(\varepsilon_{x}-\varepsilon_{2})\cot^{2}\theta$$

Because *ε*_2_ is negative, it can be expressed as follows:

(15) $$-\varepsilon_{2}=\beta \varepsilon_{0}=\frac{0.002}{0.8+170\varepsilon_{1}}\leq 0.002$$

For *ε_x_*, if the section is near the loading point, *ε_x_* = 0. If the section is near the support, because it is crossed by longitudinal reinforcement, *ε_x_* is obtained using following formula [36]:

(16) $$\varepsilon_{x}=0.5\varepsilon_{s}=T/\left(2E_{s}A_{s}\right)$$

#### 4.2.4. Parameter w

*w*, the height of the strut cross-section, has different expression and calculation methods at the support and loading points. This paper specifies the calculation methods at the support and loading point, respectively, as follows:

(1) As shown in Figure 14, *w* at support point A can be expressed as follows:

(17) $$w=h_{a}\cos\theta +l_{b}\sin\theta$$

where *h_a_* is the height of the tie cross-section, generally, $h_{a}=2(h-d)$, *d* is the effective height of the beam cross-section, and *l_b_* is the width of the base plate at the support point.

**Figure 17:**
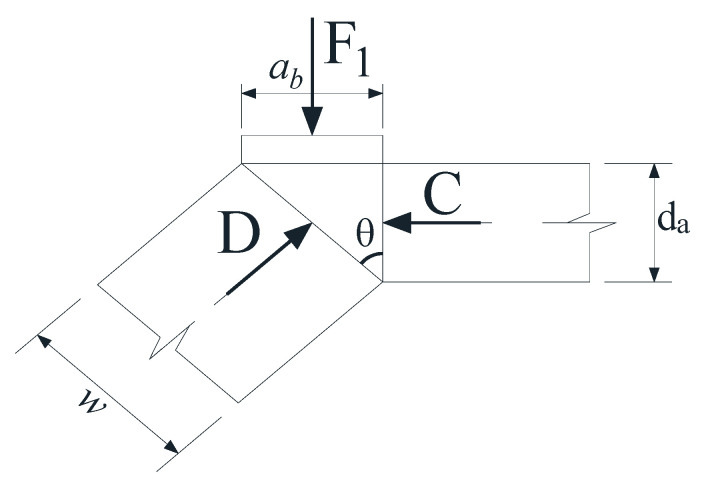
Force and geometric diagram of point C in Model 1.

(2) As shown in Figure 17, *w* at load point C can be expressed as follows:

(18) $$w=a_{b}\sin\theta +d_{a}\cos\theta$$

where *a_b_* is the width of the base plate at loading point.

#### 4.2.5. Calculation Process

Since *w* is different at the support and the loading points, the calculation process of shear capacity at the corresponding position is also different. To obtain the shear capacity of the beam, the shear capacity provided by the top (*V_t_*) and the bottom (*V_b_*) of the concrete diagonal strut must be calculated, respectively. It is noted that *V*_1_ equals the smaller of *V_t_* and *V_b_*. The calculation process of *V_t_* and *V_b_* is as follows:

(1) The shear capacity provided by the top of strut is *V*_t_.

For the top end of strut, there is no crossing of the tensile reinforcement. The effective strength of concrete is 0.85 *f_c_*′ because of C-C-C node [36,40], and Equation (3) can be expressed as follows:

(19) $$D=0.85f_{c}^{\prime }bw$$

We can combine Equations (1), (18) and (19) to obtain

(20) $$V_{t}=0.85f_{c}^{\prime }b(a_{b}sin^{2}\theta +d_{a}\sin\theta \cos\theta )$$

(2) The shear capacity provided by the bottom of strut is *V_b_*.

We can combine Equations (2), (14)–(16) to obtain

(21) $$\varepsilon_{1}=\frac{V_{b}\cot\theta }{2E_{s}A_{s}}+(\frac{V_{b}\cot\theta }{2E_{s}A_{s}}+\frac{0.002}{0.8+170\varepsilon_{1}})\cot^{2}\theta$$

and combine Equations (1), (3) and (9) to obtain

(22) $$V_{b}=\frac{f_{c}^{\prime }\sin\theta }{0.8+170\varepsilon_{1}}bw$$

We can also combine Equations (21) and (22) to obtain

(23) $$170\varepsilon_{1}^{2}+0.8\varepsilon_{1}-\frac{f_{c}^{\prime }bw\cos\theta }{2E_{s}A_{s}\sin^{2}\theta }-0.002\cot^{2}\theta =0$$

Since *ε*_1_ is the compressive strain of concrete, the solution of Equation (24) is as follows:

(24) $$\varepsilon_{1}=\frac{-0.8+\sqrt{0.64+680(\frac{f_{c}^{\prime }bw\cos\theta }{2E_{s}A_{s}\sin^{2}\theta }+0.002\cot^{2}\theta )}}{340}$$

We can then combine Equations (22) and (24) to obtain

(25) $$V_{b}=\frac{2f_{c}^{\prime }bw\sin\theta }{0.8+\sqrt{0.64+680(\frac{f_{c}^{\prime }bw\cos\theta }{2E_{s}A_{s}\sin^{2}\theta }+0.002\cot^{2}\theta )}}$$

The ultimate bearing capacity of concrete beams without web reinforcement is the failure of the top or bottom of the strut, so *V*_1_ is the smaller value of *V_t_* and *V_b_*, i.e.

(26) $$V_{1}=\min(V_{t},V_{b})$$

### 4.3. Shear Capacity Provided by Stirrups: V_2_

For Model 2, the shear capacity is similar to that provided by steel stirrups in RC beams: $V_{\mathrm{sv}}=f_{\mathrm{yv}}\frac{A_{sv}}{S}h_{0}$. The shear capacity of the beam reinforced with FRP stirrups is calculated according to Equation (27):

(27) $$V_{2}=f_{sv}\frac{A_{sv}}{S}h_{0}$$

where *f_sv_* is the effective stress of the FRP stirrup. It can be found from the test phenomenon that when the beam specimens are damaged by shear, the FRP stirrup intersecting the inclined crack does not reach its ultimate tensile strength, so *f_sv_* is taken as $0.004E_{sv}$ [18].

Substitute Equation (26) into $V=V_{1}+V_{2}$:

(28) $$V=V_{1}+V_{2}=\min(V_{b},V_{t})+0.004E_{f}\frac{A_{sv}}{S}h_{0}$$

### 4.4. Calculation Results and Discussion

At present, the design codes and guidelines used to calculate the shear capacities of FRP-reinforced concrete beams mainly include ACI 440.1R-15 [18], CSA S806:12 (reaffirmed 2021) [19], GB 50608-2020 [20], and ISIS-2007 [21]. Based on these design codes and guidelines, the shear capacity of FRP-reinforced concrete beams is divided into the shear capacity contributed by concrete (*V_c_*) and the FRP stirrups (*V_FRP_*), and these values are listed in detail in Table 4.

**Figure 18:**
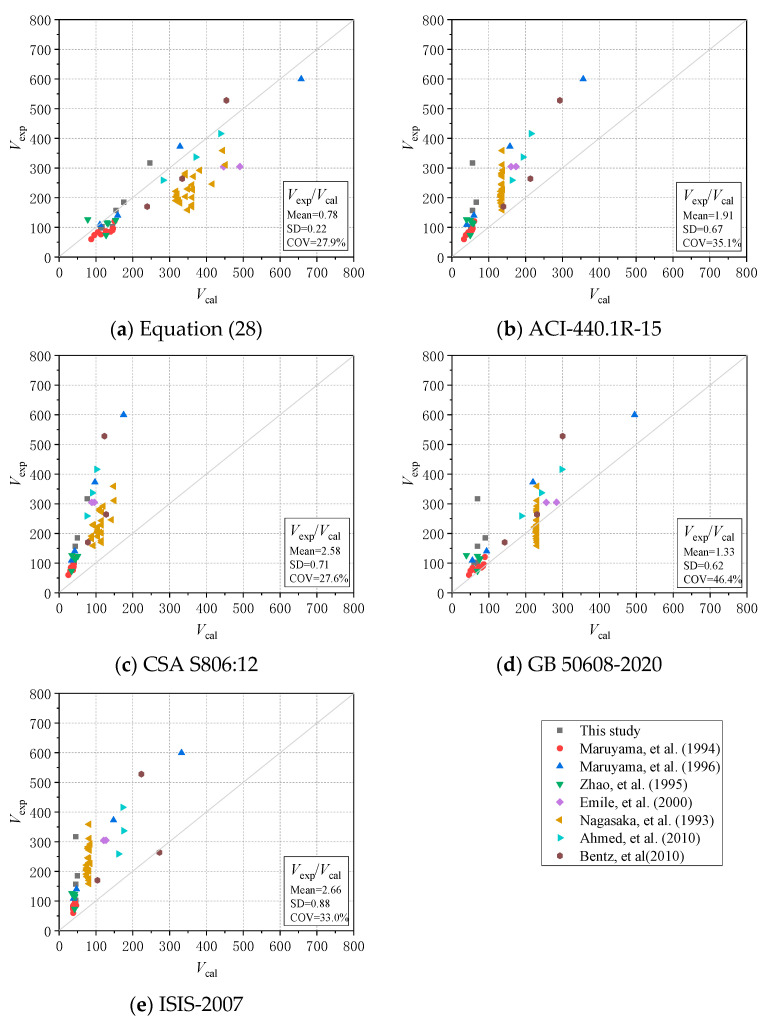
Comparison between calculation and experimental results [7,41,42,43,44,45,46].

In order to evaluate the reliability of the shear formula proposed in this paper (i.e., Equation (28)), a database containing 56 test beams was established in this study. The test parameters of 56 beam specimens, including 6 beams in this paper and 50 beams extracted from previously studies [11,41,42,43,44,45,46], are listed in Table 5. The parameters of these specimens include shear span to depth ratio *λ*, concrete axial compressive strength *f_c_*′, FRP stirrup type, and the elastic modulus of the FRP stirrup *E_sv_*_, etc._ The test results of beams reinforced with FRP stirrups were compared with the calculation results of using Equation (28) and the equation mentioned in ACI 440.1R-15, CSA S806:12 (reaffirmed 2021), GB 50608-2020, and ISIS-2007. Further, Figure 18 shows the comparison between the calculation results obtained from the three prediction formulas and the test results. The diagonal line serves as a reference to highlight the deviation between the experimentally observed shear capacity and the shear capacity calculated using the equations.

In order to verify the accuracy of the shear capacity calculation model proposed in this paper, the three indicators of the mean value (Mean), standard deviation (SD), and coefficient of variation (COV) of the comparison value *V_c_*/*V_exp_* were calculated, as shown in Table 5 and Figure 18. It can be observed that the calculation results of ACI, CSA, GB and ISIS are quite conservative. The mean values of the experimental calculations (*V_exp_/V_cal_*) using ACI, CSA, GB and ISIS are 1.91, 2.58, 1.33 and 2.66, respectively. Because the calculations of ACI, GB, and ISIS did not consider the influence of the shear–span ratio, and alongside the CSA code, we adopted lower reduction factors for both the concrete and stirrup parts, which seriously underestimated the shear capacity. For the results calculated in the GB 50608-2020, some of the shear capacity values of beam specimens with less stirrup spacing were overestimated, but the other points in Figure 18d all located not far above the diagonal line. Moreover, its calculation process is the simplest, so it has more application guidance than others.

It can be seen that the SD value and COV value of the *V_c_/V_exp_* are the smallest for Equation (28). The shear capacity calculated by Equation (28) is close to the experimental results, and the mean value and coefficient of variation COV is only 0.77 and 27.4%. It is noted that, similar to GB 50608-2020, the shear capacity of Equation (28) was also overestimated when the stirrup spacing was less than 80 mm. Combined with the experiment results, it can be seen that stirrup spacing of less than 100 mm does not obviously increase the shear capacity. Therefore, the stirrup spacing should have a lower limit of about 100 mm. The calculation results showed that Equation (28) proposed in this paper not only considered the effects of concrete, longitudinal reinforcement and FRP stirrups on shear capacity within other codes and guidelines, but was also more accurate than the models in current codes. In a word, it can be concluded that Equation (28) has a higher precision compared with the existing formulas in the prediction of the shear capacities of concrete beams reinforced with FRP stirrups. In future research, more and more test data on concrete beam reinforced with FRP stirrups should be collected to verify the accuracy and reliability of Equation (28) before it can be used in potential applications or modifications to current design codes.

## 5. Conclusions

In this study, the shear behavior of concrete beams reinforced with FRP stirrups was investigated, and the deformation and shear capacity of the beam specimens were studied. A shear capacity calculation model based on the strut-and-tie model is proposed and is compared with design codes and guidelines from a database containing 56 beam specimens. The main conclusions can be drawn from this study as follows:

(1) There is a positive linear relationship between the shear force and the mid-span deflection. The shear span to depth ratio *λ* has a significant effect on the shear–deflection response of beam specimens, and the increase in *λ* reduced the stiffness of the specimen. Compared with *λ*, stirrup spacing *S* has less influence on the ultimate load of the specimen, and when the stirrup spacing is large (*S* = 200 mm), the stiffness of the specimen decreased in the later stage of loading.
(2) The shear span to depth ratio *λ* is an important factor affecting the shear capacity and failure mode of beam specimens. When *λ* increases from 1 to 2, 3, and 4, the ultimate shear capacity of the specimen beam decreases by 50.5%, 67.7%, and 69.2%, respectively. When *λ* increases from 1 to 2 and 3, and the failure mode was changed from baroclinic failure to shear compression failure and diagonal tension failure.
(3) The shear capacity of RC concrete beams without stirrups was calculated through the strut-and-tie model, then the FRP stirrups’ influence was taken into account. Finally, a new model for calculating the shear capacity of concrete beams with FRP stirrups is established.
(4) By comparing the actual shear capacity of a large number of beams with the predicted capacity based on ACI-440.1R-15, CSA S806:12, GB 50608-2020, ISIS-2007 and Equation (28) proposed in this article, it was concluded that overall, Equation (28) is more accurate for these results than other codes, and its mean and COV values are 0.77 and 27.4%, respectively.

## Figures and Tables

**Table 1:** Details of concrete beams reinforced with GFRP stirrups.

Beam Specimen	Longitudinal Reinforcement		GFRP Stirrup			*a* (mm)	*λ*
	Top	Bottom	Diameter (mm)	*S* (mm)	*ρ_sv_* (%)		
B-1	2 ϕ 20	4 ϕ 20	6	150	0.25	240	1
B-2	2 ϕ 20	4 ϕ 20	6	150	0.25	480	2
B-3	2 ϕ 20	4 ϕ 20	6	150	0.25	720	3
B-4	2 ϕ 20	4 ϕ 20	6	150	0.25	960	4
B-5	2 ϕ 20	4 ϕ 20	6	100	0.38	480	2
B-6	2 ϕ 20	4 ϕ 20	6	200	0.19	480	2

**Table 2:** Tensile test results of GFRP bars.

Specimen No.	Maximum Tensile Force (kN)	Ultimate Tensile Strength (MPa)	Elastic Modulus (GPa)	Elongation at Break (%)
1	21.1	749.0	57.2	1.3
2	19.9	702.1	56.2	1.2
3	19.1	676.9	58.1	1.2
4	21.9	774.5	52.5	1.5
5	19.2	679.0	54.1	1.3
Mean	20.2	716.3	55.6	1.3

**Table 3:** Test results of beam specimens.

Beam Specimen	Δ*_max_* (mm)	*V_cr_* (kN)	*V_scr_* (kN)	*V_exp_* (kN)	Failure Type
B-1	17.8	35	175	317.0	DC
B-2	14.3	20	85	157.0	SC
B-3	9.4	25	60	102.5	DT
B-4	14.1	15	35	97.5	DT
B-5	16.4	20	110	185.0	SC
B-6	13.2	27	70	102.5	SC

Note: DT represents “diagonal tension failure”; DC represents “diagonal compression failure”; SC represents “shear compression failure”.

**Table 4:** List of design code or guideline equations used to calculate the shear capacities of concrete beams reinforced with FRP bar stirrups.

The Design Code or Guideline	Concrete Contribution (*V*_c_)	FRP Stirrup Contribution (*V*_FRP_)
ACI-440.1R-15 [18]	$\begin{array}{l} V_{c}=\frac{2}{5}\sqrt{f_{c}\prime }b_{w}(kd)\\ k=\sqrt{2\rho n+{\left(\rho n\right)}^{2}}-\rho n\\ n=E_{s}/E_{c} \end{array}$	$\begin{array}{l} V_{FRP}=\frac{A_{fv}f_{fv}d}{s}\\ f_{fv}=0.004E_{sv}\leq f_{fb}\\ f_{fb}=(0.05\frac{r_{b}}{d_{b}}+0.3)f_{fu}\leq f_{fu} \end{array}$
CSA S806:12 (reaffirmed 2021) [19]	$\begin{array}{l} V_{c}=0.05\lambda \phi_{c}k_{m}k_{r}k_{\alpha }k_{s}{\left(f_{c}^{\prime }\right)}^{\frac{1}{3}}b_{w}d_{v}\\ k_{m}=\sqrt{\frac{V_{f}d}{M_{f}}}\leq 1.0\\ k_{r}=1+{\left(E_{s}\rho \right)}^{\frac{1}{3}}\\ 1.0\leq k_{\alpha }=\frac{2.5}{\frac{M_{f}}{V_{f}}d}\leq 2.5\\ k_{s}=\frac{750}{450+d}\leq 1.0\\ 0.11\varphi_{c}\sqrt{f_{c}^{\prime }}b_{w}d_{v}\leq \frac{V_{c}}{k_{\alpha }k_{s}}\leq 0.22\varphi_{c}\sqrt{f_{c}^{\prime }}b_{w}d_{v}\\ d_{v}=\max(0.9d,0.72h)\\ \phi_{c}=0.65\\ \lambda =1.0 \end{array}$	$\begin{array}{l} V_{FRP}=\frac{0.4\varphi_{F}A_{fv}f_{fu}d_{v}}{s}\cot\theta \\ 30{}^{\circ}\leq \theta =30{}^{\circ}+7000\varepsilon_{1}\leq 60{}^{\circ}\\ \varepsilon_{1}=\frac{V_{f}(\frac{a}{d_{v}}+1)}{2E_{s}A_{s}}\\ f_{fu}\leq 0.005E_{f}\\ \varphi_{F}=0.75 \end{array}$
GB 50608-2020 [20]	$\begin{array}{l} V_{c}=0.86f_{t}b_{w}kd\\ k=\sqrt{2\rho \alpha_{f}+{\left(\rho \alpha_{f}\right)}^{2}}-\rho \alpha_{f} \end{array}$	$\begin{array}{l} V_{FRP}=\frac{A_{fv}f_{fv}h_{0f}}{s}\\ A_{fv}=nA_{fv1}\\ f_{fv}=\min(0.004E_{f},f_{f_{bend}})\\ f_{f_{bend}}=(0.3+0.05\frac{r_{v}}{d_{v}})f_{fd} \end{array}$
ISIS-2007 [21]	$\begin{array}{l} V_{c}=0.2\lambda \phi_{c}\sqrt{f_{c}^{\prime }}b_{w}d\sqrt{\frac{E_{S}}{E_{steel}}}\\ \lambda =1.0\\ \mathrm{For}\;\mathrm{concrete}-\mathrm{cast}-\mathrm{in}-\mathrm{situ},\;\phi_{c}=0.6 \end{array}$	$\begin{array}{l} V_{FRP}=\phi_{frp}\frac{A_{sv}\sigma_{v}d_{v}\cot\theta }{s}\\ \sigma_{v}=\frac{(0.05(\frac{r_{b}}{d_{s}})+0.3)f_{fv}}{1.5}\\ \mathrm{Or},\;\sigma_{v}=E_{frpv}\varepsilon_{v}\\ \varepsilon_{v}=0.0001{\left(f_{c}^{\prime }\frac{\rho E}{\rho_{sv}E_{sv}}\right)}^{0.5}\left\{1+2(\frac{\sigma_{N}}{f_{c}^{\prime }})\right\}\leq 0.0025\\ d_{v}=0.9d\\ \phi_{frp}=0.75 \end{array}$

**Table 5:** Summary of calculation results based on Equation (28), ACI-440.1R-15, CSA S806:12 (reaffirmed 2021), GB 50608-2020 and ISIS-2007 and test results of 56 beam specimens reinforced with FRP stirrups.

Resource	Specimen ID	Geometrical Characteristics				Concrete	Long. Reinforcement			Shear Reinforcement				*V*_exp_ (kN)	*V*_cal_ (kN)					*V*_exp_/*V*_cal_				
		*λ*	*h* (mm)	*d* (mm)	*b* (mm)	*f*_c_′ (MPa)	Type	*ρ*_s_ (%)	*E*_s_ (GPa)	Type	*s* (mm)	*ρ*_sv_ (%)	*E*_sv_ (GPa)		Equation (28)	ACI	CSA	GB	ISIS	Equation (28)	ACI	CSA	GB	ISIS
This article	B-1	1.0	300	240	150	26.07	Steel	2.79	200	GFRP	150	0.25	55.6	317	247	56	77	69	45	1.29	5.68	4.14	4.57	7.01
	B-2	2.0	300	240	150	26.07	Steel	2.79	200	GFRP	150	0.25	55.6	157	155	56	44	69	45	1.02	2.81	3.58	2.26	3.47
	B-3	3.0	300	240	150	26.07	Steel	2.79	200	GFRP	150	0.25	55.6	102.5	116	56	37	69	45	0.88	1.84	2.75	1.48	2.27
	B-4	4.0	300	240	150	26.07	Steel	2.79	200	GFRP	150	0.25	55.6	97.5	113	56	37	69	45	0.86	1.75	2.63	1.40	2.16
	B-5	2.0	300	240	150	26.07	Steel	2.79	200	GFRP	100	0.38	55.6	185	175	66	50	90	50	1.06	2.79	3.73	2.05	3.73
	B-6	2.0	300	240	150	26.07	Steel	2.79	200	GFRP	200	0.19	55.6	102.5	145	51	41	60	43	0.71	2.01	2.48	1.71	2.37
Maruyama et al. (1994) [41]	FF1-20	3.0	300	250	150	36.2	CFRP	0.55	94	CFRP	200	0.12	94	60.3	87	33	25	46	38	0.69	1.85	2.39	1.31	1.58
	FF2-10	3.0	300	250	150	33.1	CFRP	1.1	94	CFRP	100	0.24	94	90.3	145	55	38	84	44	0.62	1.65	2.40	1.07	2.06
	FF1-10	3.0	300	250	150	38.3	CFRP	0.55	94	CFRP	100	0.24	94	85.3	139	50	32	80	46	0.61	1.72	2.69	1.07	1.85
	FF3-10	3.0	300	250	150	31.3	CFRP	1.39	94	CFRP	100	0.24	94	96.3	146	56	39	86	43	0.66	1.71	2.44	1.12	2.24
	FF1-20	3.0	300	250	150	35	CFRP	1.06	94	CFRP	200	0.12	94	74.1	95	38	30	50	38	0.78	1.97	2.46	1.48	1.97
	FF4-10	3.0	300	250	150	30.5	CFRP	2.11	94	CFRP	100	0.24	94	120.8	150	60	43	89	43	0.80	2.00	2.78	1.35	2.83
	FF4-10	3.0	300	250	150	30.5	CFRP	2.11	94	CFRP	130	0.18	94	87.3	127	53	40	74	39	0.69	1.66	2.20	1.19	2.22
	FF4-16	3.0	300	250	150	31.3	CFRP	2.11	94	CFRP	160	0.15	94	76.3	113	48	37	64	38	0.67	1.59	2.04	1.19	2.02
	FF4-20	3.0	300	250	150	34.9	CFRP	2.11	94	CFRP	200	0.12	94	83.8	105	45	36	56	38	0.80	1.88	2.31	1.51	2.23
Maruyama et al. (1996) [42]	#25/26	2.5	300	250	150	34	CFRP	1.04	100	GFRP	120	0.43	30	109.2	111	40	34	55	39	0.99	2.71	3.24	1.97	2.79
	#27/28	2.5	300	250	150	34	CFRP	1.04	100	GFRP	60	0.86	30	140.2	159	60	42	94	47	0.88	2.35	3.31	1.49	2.97
	#30	2.5	550	500	300	29.5	CFRP	1.04	100	GFRP	240	0.43	30	372.8	328	157	97	220	148	1.14	2.37	3.84	1.70	2.52
	#32	2.5	800	750	450	29.5	CFRP	1.07	100	GFRP	360	0.43	30	599.3	657	356	175	495	332	0.91	1.68	3.43	1.21	1.80
Zhao et al. [43]	#10	3.0	300	250	150	34.3	CFRP	3.03	105	GFRP	90	0.42	39	113.7	133	58	44	76	42	0.86	1.97	2.58	1.50	2.69
	#14	3.0	300	250	150	34.3	CFRP	3.03	105	CFRP	90	0.42	10	126.6	78	40	35	39	34	1.63	3.20	3.65	3.24	3.67
	#16	3.0	300	250	150	34.3	CFRP	2.27	105	GFRP	90	0.42	39	116.9	129	54	42	73	42	0.90	2.16	2.80	1.61	2.77
	#18	2.0	300	250	150	34.3	CFRP	1.51	105	GFRP	90	0.42	39	124	154	50	51	69	42	0.81	2.50	2.45	1.79	2.94
	#19	4.0	300	250	150	34.3	CFRP	1.51	105	GFRP	90	0.42	39	73.8	127	50	34	69	42	0.58	1.49	2.17	1.07	1.75
Emile et al. [44]	CC-3	3.2	560	470	135	50	CFRP	1.25	137	CFRP	157	0.36	137	305	491	174	96	283	127	0.62	1.75	3.19	1.08	2.40
	CG-3	3.2	560	470	135	50	CFRP	1.25	137	GFRP	157	1.07	41	304.5	446	160	89	256	121	0.68	1.90	3.44	1.19	2.51
Nagasaka et al. [45]	AC0560M	1.2	300	253	250	28.9	AFRP	1.9	56	CFRP	80	0.5	80	246.2	416	135	142	230	79	0.59	1.82	1.73	1.07	3.13
	AC1060M	1.2	300	253	250	34	AFRP	1.9	56	CFRP	40	1	40	311	452	137	150	231	82	0.69	2.27	2.08	1.35	3.81
	AC1560M	1.2	300	253	250	32.9	AFRP	1.9	56	CFRP	27	1.48	27	359	444	137	148	231	81	0.81	2.63	2.42	1.56	4.43
	AC0590M	1.8	300	253	250	28.9	AFRP	1.9	56	CFRP	80	0.5	80	204	343	135	111	230	79	0.59	1.51	1.84	0.89	2.59
	AC1090M	1.8	300	253	250	28.9	AFRP	1.9	56	CFRP	40	1	40	276.6	343	135	111	230	79	0.81	2.04	2.50	1.20	3.52
	AC1590M	1.8	300	253	250	28.9	AFRP	1.9	56	CFRP	27	1.48	27	282.5	343	135	111	230	79	0.82	2.09	2.55	1.23	3.59
	AC0512M	2.4	300	253	250	32.9	AFRP	1.9	56	CFRP	80	0.5	80	158.9	349	137	92	231	81	0.46	1.16	1.72	0.69	1.96
	AC1012M	2.4	300	253	250	32.9	AFRP	1.9	56	CFRP	40	1	40	229.6	349	137	92	231	81	0.66	1.68	2.49	1.00	2.83
	AA0590M	1.8	300	253	250	33.5	AFRP	1.9	56	AFRP	80	0.5	80	201.1	361	137	115	231	81	0.56	1.47	1.75	0.87	2.47
	AA1090M	1.8	300	253	250	34.7	AFRP	1.9	56	AFRP	40	1	40	271.7	365	137	116	231	82	0.74	1.98	2.34	1.18	3.31
	AH0590M	1.8	300	253	250	33.5	AFRP	1.9	56	G/CFRP	80	0.5	80	169.7	361	137	115	231	81	0.47	1.24	1.48	0.73	2.08
	AH1090M	1.8	300	253	250	33.5	AFRP	1.9	56	G/CFRP	40	1	40	243.3	361	137	115	231	81	0.67	1.78	2.12	1.05	2.99
	AG0590M	1.8	300	253	250	33.5	AFRP	1.9	56	G/CFRP	80	0.5	80	175.6	361	137	115	231	81	0.49	1.28	1.53	0.76	2.16
	AG1090M	1.8	300	253	250	33.5	AFRP	1.9	56	G/CFRP	40	1	40	228.6	361	137	115	231	81	0.63	1.67	1.99	0.99	2.81
	AC1090L	1.8	300	253	250	23.5	AFRP	1.9	56	CFRP	40	1	40	207	321	133	105	229	75	0.64	1.55	1.97	0.90	2.75
	AC1590L	1.8	300	253	250	22.6	AFRP	1.9	56	CFRP	27	1.48	27	221.7	317	133	104	224	75	0.70	1.67	2.13	0.99	2.98
	AC1012L	2.4	300	253	250	24.3	AFRP	1.9	56	CFRP	40	1	40	182.5	328	134	89	229	76	0.56	1.37	2.05	0.80	2.41
	AC1512L	2.4	300	253	250	23	AFRP	1.9	56	CFRP	27	1.48	27	191.3	325	133	88	229	75	0.59	1.44	2.17	0.84	2.56
	AA1090L	1.8	300	253	250	22.6	AFRP	1.9	56	AFRP	40	1	40	190.3	317	133	104	229	75	0.60	1.43	1.82	0.83	2.55
	AA1590L	1.8	300	253	250	22.6	AFRP	1.9	56	AFRP	27	1.48	27	203.1	317	133	104	229	75	0.64	1.53	1.95	0.89	2.73
	AH1090L	1.8	300	253	250	23.5	AFRP	1.9	56	G/CFRP	40	1	40	190.3	321	133	105	229	75	0.59	1.43	1.81	0.83	2.53
	AH1590L	1.8	300	253	250	23.5	AFRP	1.9	56	G/CFRP	27	1.48	27	211.9	321	133	105	229	75	0.66	1.59	2.01	0.93	2.82
	AC1590M′	1.8	300	253	250	39.5	AFRP	1.9	56	CFRP	27	1.48	27	292.3	382	138	120	231	85	0.76	2.11	2.44	1.27	3.45
	AC1512M′	2.4	300	253	250	39.2	AFRP	1.9	56	CFRP	27	1.48	27	226.6	363	138	94	231	85	0.62	1.64	2.40	0.98	2.68
Ahmed et al. [46]	SG-9.5-2	3.3	700	615	180	39.5	STEEL	1.51	200	GFRP	300	0.262	45	259	282	163	75	188	161	0.92	1.59	3.45	1.38	1.61
	SG-9.5-3	3.3	700	615	180	41	STEEL	1.51	200	GFRP	200	0.394	45	337	371	192	90	242	175	0.91	1.75	3.75	1.39	1.93
	SG-9.5-4	3.3	700	615	180	33.5	STEEL	1.51	200	GFRP	150	0.526	45	416	438	215	101	298	172	0.95	1.94	4.11	1.40	2.41
Bentz et al. [11]	L05-1	3.3	1000	937	450	46	GFRP	0.51	37	GFRP	400	0.09	41	264	334	213	128	231	272	0.79	1.24	2.07	1.14	0.97
	L20-1	3.6	1000	857	450	36	GFRP	2.23	37	GFRP	400	0.09	41	527.9	454	293	123	300	223	1.16	1.80	4.29	1.76	2.37
	M20-1	7.5	500	405	450	35	GFRP	2.36	37	GFRP	400	0.09	41	170.3	239	140	78	143	104	0.71	1.22	2.18	1.19	1.64
Mean	-	-	-	-	-	-	-	-	-	-	-	-	-	-	-	-	-	-	-	0.77	1.91	2.58	1.33	2.66
SD	-	-	-	-	-	-	-	-	-	-	-	-	-	-	-	-	-	-	-	0.21	0.67	0.71	0.62	0.88
COV (%)	-	-	-	-	-	-	-	-	-	-	-	-	-	-	-	-	-	-	-	27.4	35.1	27.6	46.4	33.0

Note: SD represents “standard deviation”; COV represents “coefficient of variation”; “-” represents “not available”.

## Data Availability

Data are contained within the article.

## Nomenclature

*a*	Horizontal distance from loading point to supporting point (mm)
*λ*	Shear span to depth ratio
*S*	Stirrup spacing (mm)
*ρ_sv_*	Stirrup reinforcement ratio (%)
*ρ*	Longitudinal reinforcement ratio (%)
*V_cr_*	First crack load (kN)
*V_scr_*	First diagonal shear crack (kN)
*V_exp_*	Shear capacity of experiment (kN)
*ω_max_*	Maximum diagonal crack width of beam (mm)
*Δ_max_*	Maximum deflection (mm)
*V*_1_	Shear capacity of concrete beams without web reinforcement (kN)
*θ*	Angle between strut and beam transverse axis (in degree)
*D*	Compression capacity of strut (kN)
*f*_2*max*_	Compressive stress of strut (kN)
*f_cu_*	Standard value of the cubic compressive strength of concrete
*f_c_′*	Specified compressive strength of concrete (MPa)
*β*	Softening effect coefficient of the compressive strength of cracked concrete
*ε*_0_	Maximum compressive strain of softened concrete
*ε_c_*	Compressive strain of softened concrete
*σ_c_*	Compressive stress of softened concrete (MPa)
*ε*_1_	Principal tensile strain of concrete in the direction perpendicular to strut
*ε*_2_	Principal compressive strain of strut
*ε_x_*	Longitudinal strain of element
*ε_t_*	Transverse strain of element
*γ_xt_*	Shear strain of element
*w*	Height of strut cross-section (mm)
*h*	Height of beam cross-section (mm)
*h_a_*	Height of tie cross-section (mm)
*d*	Distance from the extreme compression fibre to the centroid of longitudinal tension force (mm)
*l_b_*	Width of base plate at support point (mm)
*a_b_*	Width of base plate at load point (mm)
*d_a_*	Height of concrete horizontal compression bar between nodes C and D (mm)
*k*	Height coefficient of concrete compression area
*n*	Ratio of elastic modulus of longitudinal reinforcement to elastic modulus of concrete
*E_s_*	Elastic modulus of longitudinal reinforcement (MPa)
*E_c_*	Elastic modulus of concrete (MPa)
*V_t_*	Shear capacity provided by the top of strut (kN)
*V_b_*	Shear capacity provided by the bottom of strut (kN)
*V*_2_	Shear capacity provided by stirrups (kN)
*f_sv_*	Effective stress of FRP stirrup (MPa)
*E_sv_*	Elastic modulus of FRP stirrup (MPa)
*V_cal_*	Shear capacity of calculation (kN)
